# Epilepsy: A Study of the Idiopathic Disease

**Published:** 1908-09

**Authors:** 


**Epilepsy: A Study of the Idiopathic Disease.** By William Aldren Turner, M.D. Pp. xi., 272. London: Macmillan & Co. 1907.

This book can be cordially recommended as giving an excellent account of the modern views on epilepsy. Dr. Turner, during his long experience at the Chalfont Colony for Epileptics, and at the National Hospital in Queen Square, has had exceptional opportunities for studying the disease, and in this work are embodied his mature conclusions. The reader will, for one thing, learn from it that the outlook is not so hopeless in epilepsy as is often thought; that a considerable percentage of cases show an arrest of the disease under appropriate measures of treatment, and for a further number, in whom this fortunate result is not obtained, much may be done to mitigate their affliction and provide them with suitable occupations. There is no doubt that the "colony" treatment marks a very distinct advance in dealing with epileptics, and in ameliorating their lives.

The book gives a very thorough clinical study of epilepsy. The chapters on the mental changes which occur in the disease, and on prognosis, are particularly valuable. The pathological basis of the affection, so far as it is at present known, receives entirely adequate treatment. In the author's opinion, the immediate cause of the fits is cortical stasis, resulting from obstruction to the blood supply by intravascular clotting. The book concludes with a full account of the treatment of epilepsy, which will be found very useful, and an appendix on Epileptic Colonies, which contains much valuable information.

